# Editorial: Dietary practices, food consumption and nutritional status of children and adolescents in Latin America and the Caribbean

**DOI:** 10.3389/fpubh.2023.1248337

**Published:** 2023-07-26

**Authors:** Juliana Souza Oliveira, Risia Cristina Egito de Menezes, Larissa Loures Mendes

**Affiliations:** ^1^Nutrition Unit, Vitória Academic Center, Federal University of Pernambuco, Vitória de Santo Antão, Brazil; ^2^College of Nutrition, Federal University of Alagoas, Maceió, Brazil; ^3^Nutrition Department, Federal University of Minas Gerais, Belo Horizonte, Brazil

**Keywords:** children, adolescents, eating habits, food consumption, obesity, intervention measures

Radical changes in the global food system, and consequently in food environments, economic status, and lifestyle, have established new paradigms and changes in food practices and choices (1–3). These play an essential role in the nutritional status of individuals, especially children, and adolescents, due to the increased nutritional needs generated by rapid growth and development. Nutritional disorders during childhood and adolescence are problems that require attention due to the repercussions they cause to health (4).

These factors point to the need for a better understanding of the issues related to food consumption in this population (5, 6), which includes reflecting on the impact of the pandemic of COVID-19 on the behavioral changes of children and adolescents (7). In addition, there is an observed reduction in physical activity levels and more time spent in sedentary activities such as watching television and using computers, video games, cell phones, and tablets (8, 9).

Food consumption is one of the main factors for the precocious appearance and early onset of nutritional disorders in all their forms, especially anemia and growth retardation, among other non-communicable chronic diseases (NCDs) (10, 11). However, food is considered a modifiable behavioral factor, highlighting childhood as a window of opportunity to promote proper and healthy eating, an investment that can positively impact health throughout life.

Despite this consolidated scientific evidence, global efforts to promote good and healthy eating to prevent future health complications were unsuccessful in this generation. On the contrary, there has been an accelerated increase in malnutrition across the planet and income strata. In 2020, the United Nations International Children's Emergency Fund (UNICEF) estimated that 340 million children under five still suffer from vitamin deficiencies and other micronutrients essential for growth (12).

In Latin America, between 42.5 and 51.8 million children and adolescents are overweight or living with obesity. This number represents about 20–25% of the population. Many countries in this region face a double burden with the simultaneous manifestation of malnutrition (micronutrient deficiencies, low weight, and short height) and overweight. This scenario is common among children and children and adolescents with overweight or obesity (13).

Research on this Topic, “*Dietary practices, food consumption and nutritional status of children and adolescents in Latin America and the Caribbean*”, presents data, reflections, and discussions on food access profiles, food environment, dietary practices and patterns, food consumption, nutritional status, and changes in food consumption during the COVID-19 pandemic among children and adolescents. In addition, there are studies on the interventions in Latin American and Caribbean countries, particularly Chile and Brazil.

Data presented here is undeniably relevant for planning, implementing, and evaluating public policies that contribute to healthier eating for children and adolescents. Such policies can also ensure access to food of a nutritional quality that is socially supported (14), based on interventions valid for each context, in Chile and Brazil. Also, data is crucial to developing more specific actions, considering the family, school, and primary healthcare contexts.

It is essential to note that to tackle the current epidemic of obesity and the consumption of ultra-processed foods (UPF), Swinburn et al. (3) recommend: the adoption of taxation of these foods, prominent nutrition labeling of UPFs, regulation of advertising of UPFs; and promotion of environments food with an emphasis on organizational settings such as schools. There is also a need for multi-component interventions for the prevention and treatment of obesity and NCDs among children and adolescents, enabling a greater understanding of the multiple components that act on these individuals' eating behavior and allowing more effective intervention. In this context, the school is a privileged place where these individuals spend much of their day (15, 16).

Thus, individual responsibility can only have full effect when people have access to a healthy lifestyle. Moreover, at the societal level, it is essential to sustain the implementation of evidence-based policies that enable children and adolescents to achieve a regularly active life and that healthy foods are available and easily accessible.

The articles on this Research Topic are indispensable for those who study nutritional status, practices, patterns, and food consumption among children and/or adolescents. Similarly, the quality of the data obtained on the diet and nutrition of children and adolescents allows a glimpse into future studies that may enable measures of care, prevention, and control of nutritional problems that begin in childhood and adolescence and may persist into adulthood.

## Author contributions

JSO, RCEM, and LLM contributed to conception of the study. JSO wrote the first draft of the manuscript. All authors contributed to manuscript revision, read, and approved the submitted version.
